# Transcutaneous Vagus Nerve Stimulation: Retrospective Assessment of Cardiac Safety in a Pilot Study

**DOI:** 10.3389/fpsyt.2012.00070

**Published:** 2012-08-07

**Authors:** Peter M. Kreuzer, Michael Landgrebe, Oliver Husser, Markus Resch, Martin Schecklmann, Florian Geisreiter, Timm B. Poeppl, Sarah Julia Prasser, Goeran Hajak, Berthold Langguth

**Affiliations:** ^1^Department of Psychiatry and Psychotherapy, University of RegensburgRegensburg, Germany; ^2^Klinik und Poliklinik für Innere Medizin II, University of Regensburg Medical CenterRegensburg, Germany; ^3^Department of Psychiatry, Psychosomatic Medicine and Psychotherapy, Social Foundation BambergBamberg, Germany

**Keywords:** vagus nerve, neuromodulation, tinnitus, ECG, electrocardiography, cardiac arrhythmia

## Abstract

**Background:** Vagus nerve stimulation has been successfully used as a treatment strategy for epilepsy and affective disorders for years. Transcutaneous vagus nerve stimulation (tVNS) is a new non-invasive method to stimulate the vagus nerve, which has been shown to modulate neuronal activity in distinct brain areas. **Objectives:** Here we report effects of tVNS on cardiac function from a pilot study, which was conducted to evaluate the feasibility and safety of tVNS for the treatment of chronic tinnitus. **Methods:** Twenty-four patients with chronic tinnitus underwent treatment with tVNS over 3–10 weeks in an open single-armed pilot study. Safety criteria and practical usability of the neurostimulating device were to investigate by clinical examination and electrocardiography at baseline and at several visits during and after tVNS treatment (week 2, 4, 8, 16, and 24). **Results:** Two adverse cardiac events (one classified as a severe adverse event) were registered but considered very unlikely to have been caused by the tVNS device. Retrospective analyses of electrocardiographic parameters revealed a trend toward shortening of the QRS complex after tVNS. **Conclusion:** To our knowledge this is one of the first studies investigating feasibility and safety of tVNS in a clinical sample. In those subjects with no known pre-existing cardiac pathology, preliminary data do not indicate arrhythmic effects of tVNS.

## Introduction

Electrical stimulation of the vagus nerve (VNS) is an FDA-approved therapy tool for both refractory depression and epilepsy (Schachter and Saper, 1998; Ben-Menachem, 2002; Grimm and Bajbouj, 2010). Furthermore, it has recently emerged as a promising therapeutic approach for cardiac diseases (Schwartz et al., 2008; De Ferrari et al., 2009, 2011). It broadly affects various parts of the brain including the thalamus, cerebellum, orbitofrontal cortex, limbic system, hypothalamus, and medulla (Chae et al., 2003; Pardo et al., 2008; Vonck et al., 2008; Kosel et al., 2011). Recently vagus nerve stimulation paired with auditory stimuli has been shown to reverse pathological and behavioral changes in an animal model of tinnitus (Engineer et al., 2011). Traditionally, vagus nerve stimulation has been performed by the implantation of a neurostimulating device connected to an electrode located along the cervical branch of the vagus nerve. In order to minimize adverse effects of this procedure such as coughing during stimulation, croakiness, general operational and anesthesiological risks, and high costs, a new non-invasive neurostimulating device has been developed for transcutaneous stimulation of the afferent auricular branch of the vagus nerve (ABVN) located medial of the tragus at the entry of the acoustic meatus (tVNS^®^). The tVNS^®^ device received CE approval in 2010 (CE1275). CE marking is an indication that a medical device complies with essential health and safety requirements.

Transcutaneous vagus nerve stimulation (tVNS) targets the cutaneous receptive field of the ABVN at the outer ear (Ellrich, 2011). The human outer ear is supplied by three sensory nerves, namely the auriculotemporal nerve, the great auricular nerve, and the ABVN (Peuker and Filler, 2002). On 14 human ears the complete course of nerve supply was exposed and each branch was defined by identifying its origin. In all specimens the ABVN was found to significantly supply the cavity of conchae and exclusively supply the cymba conchae, which served as stimulation site in the present study (Peuker and Filler, 2002).

In respect to neuroimaging techniques two functional magnetic resonance imaging (fMRI) studies have been done investigating the effect of tVNS applied to the left tragus area in a sample of four healthy male adults (Dietrich et al., 2008) and to the left outer ear canal in a sample of 22 healthy volunteers (Kraus et al., 2007). Stimulation of the earlobe served as control condition. Both fMRI studies showed blood oxygen level-dependent (BOLD) signal changes. Earlobe stimulation as a sham control intervention did not exert similar effects (Kraus et al., 2007). No significant effects on heart rate, blood pressure, or peripheral microcirculation could be detected during the stimulation procedure (Kraus et al., 2007). The brain activation pattern under tVNS clearly shares features with changes observed during invasive VNS (Chae et al., 2003; Kraus et al., 2007).

In a pilot study we aimed to assess feasibility and safety of tVNS in a clinical sample of patients suffering from subjective and chronic tinnitus.

Since efferent fibers of the vagus nerve modulate cardiac function, cardiac safety has always been a concern in the therapeutic use of vagus nerve stimulation (Cristancho et al., 2011). Efferent vagal fibers to the heart are supposed to be located on the right side (Nemeroff et al., 2006). In order to avoid cardiac side effects, electrode placement is performed on the left side in treatment of central nervous diseases (Nemeroff et al., 2006).

Even if tVNS stimulates selectively afferent vagus nerve fibers, a potential reflectory effect on efferent vagus nerve function cannot be excluded. Therefore we performed electrocardiography (ECG) in all patients at baseline and during tVNS treatment.

## Materials and Methods

### Study design and subjects

The present study was conducted as a feasibility and safety study with an open, single-armed pilot study design. Clinical visits were planned according to the study protocol at screening, baseline, week 2, week 4, week 8, week 16, and week 24 (end of treatment) with a further follow-up 4 weeks after termination of the stimulation in week 28.

The study was approved by the Ethics Committee of the University of Regensburg. All study procedures were conducted in accordance with the last revision of the Declaration of Helsinki. All participants gave written informed consent after a comprehensive explanation of the procedures.

After occurrence of two cardiologic events [one newly diagnosed left bundle branch block (LBBB) and one sinusarrhythmic episode], these two cases were analyzed in detail. Moreover, ECG data from all patients in the whole sample were retrospectively analyzed at that time point.

### Patients and recruitment

The present study included 24 patients [10 male, 14 female; 59.0 ± 10.7 years (38.4–72.8)] with chronic tinnitus [167.0 ± 134.7 months duration (8.1–479.0)]. The trial was registered in an international database (ClinicalTrials.gov. identifier: NCT01176734). Patients with moderate to severe tinnitus defined by a Tinnitus Questionnaire (TQ) score >30 were recruited between May and August 2010 at the Interdisciplinary Tinnitus Center of the University of Regensburg. The mean TQ in the study sample was 49.7 ± 11.1 (32–75).

Patients with severe internal, neurological, or psychiatric comorbidities were excluded from the study. Exclusion criteria were checked by taking a detailed medical history by experienced psychiatrists. Asthma was considered a contraindication (because of theoretical risk of impairment due to a parasympathetic enhancement by VNS) as well as the permanent use of left-sided hearing aids and/or tinnitus masking devices.

### tVNS neurostimulating device

A tVNS instrument consisting of two titan electrodes mounted on a gel frame and connected to a wired neurostimulating device (CM02, Cerbomed, Erlangen, Germany) was used.

The clinical efficacy of VNS requires activation of thick myelinated afferent fibers of the vagus nerve (Vonck et al., 2007). The fibers of a sensory peripheral nerve such as the ABVN mediate touch sensation. Consequently, the stimulus intensity of tVNS was individually adjusted to a level above the individual’s detection threshold and clearly below the individual’s pain threshold. The tVNS^®^ device offered a stimulus intensity between 0.1 and 10 mA with a stimulation frequency of 25 Hz. Stimulation was active for 30 s, followed by a break of 180 s.

In addition, stimulation intensity was applied in an adjustable way by the patients according to the situational circumstances. Patients were instructed individually in the usage of the tVNS device and were recommended to use the stimulator only during daytime for safety reasons. They were instructed to adjust the stimulation intensity in order to achieve a tingling sensation that should be perceived with no painful percept at all. Every change in stimulation parameters was logged by the stimulation device. In 22 of the 24 stimulators, we could compile a detailed history of stimulation, in the remaining two cases this was not possible due to technical problems. During the study, patients used the stimulation device 24.0 ± 19.3 days with an average of 5.15 ± 1.80 h a day. The mean intensity of stimulation in the whole sample was 3.2 ± 2.5 mA.

### Electrocardiography

Twelve-electrode ECGs were recorded with a standard ECG device (Corina, GE Medical Systems Information Technology Inc., Milwaukee, USA) using GE Cardiosoft Software of General Electric Company, New York, USA. During ECG all patients were laying in supine position with the tVNS stimulator turned off.

### Statistical analyses

All continuous data were displayed as the mean with the standard deviation and were compared using the paired Student’s *t*-test or the two sample Student’s *t*-test when appropriate. In addition we reported the effect sizes d for these contrasts to estimate the clinical significance according to Cohen (*d* = 0.2 small, 0.5 medium, 0.8 high; Cohen, 1988). Statistical analyses were done in a two-steps-approach for the whole sample and for the sample without the two patients with adverse events. Statistical significance was assumed for a *p*-value < 0.05.

## Results

After occurrence of two cardiac events (one newly diagnosed LBBB and one sinusarrhythmic episode) the two cases were analyzed in detail. In addition, we conducted an analysis of all ECG recordings performed during the course of the study up to this time point as an interim analysis, in order to address safety issues.

For the whole sample missing values for visit 2 (55%) and 3 (82%) were high; thus, we concentrated on contrasts between screening/baseline (5% = 1 value missing for QRS), visit 1 (14% missing), and last available visit (32% missing). In two patients cardiac adverse events during tVNS had occurred.

A 62 years old female (patient 1) developed a palpitation episode that was monitored and treated in an intensive care unit. This event was therefore classified as a severe adverse event and reported immediately to the sponsor and the local ethics committee. The patient had noticed palpitation on a hot Saturday in the summer of 2010 that worsened in the course of the day and led to consultation of a physician in the evening. She was initially supplied with Calcium and Magnesium formula but when the complaints persisted even on the following day she sought help in a hospital and was initially observed in an intensive care unit after administration of nitroglycerine inhaler. After normal results were obtained at echocardiography, duplex sonography of the carotids, 24 h ECG monitoring, and thoracal X-ray she was dismissed from hospital without complaints 1 day later. Elevation of the dosage of her beta-blocking antiarrhythmic drug from 2.5 mg b.i.d. to 5 mg b.i.d. was recommended. Further exploration revealed that the patient had suffered comparable events earlier in the past but in-patient-treatment had not been necessary so far. tVNS treatment had been stopped immediately in this patient and she was retracted from the study.

**Figure 1:**
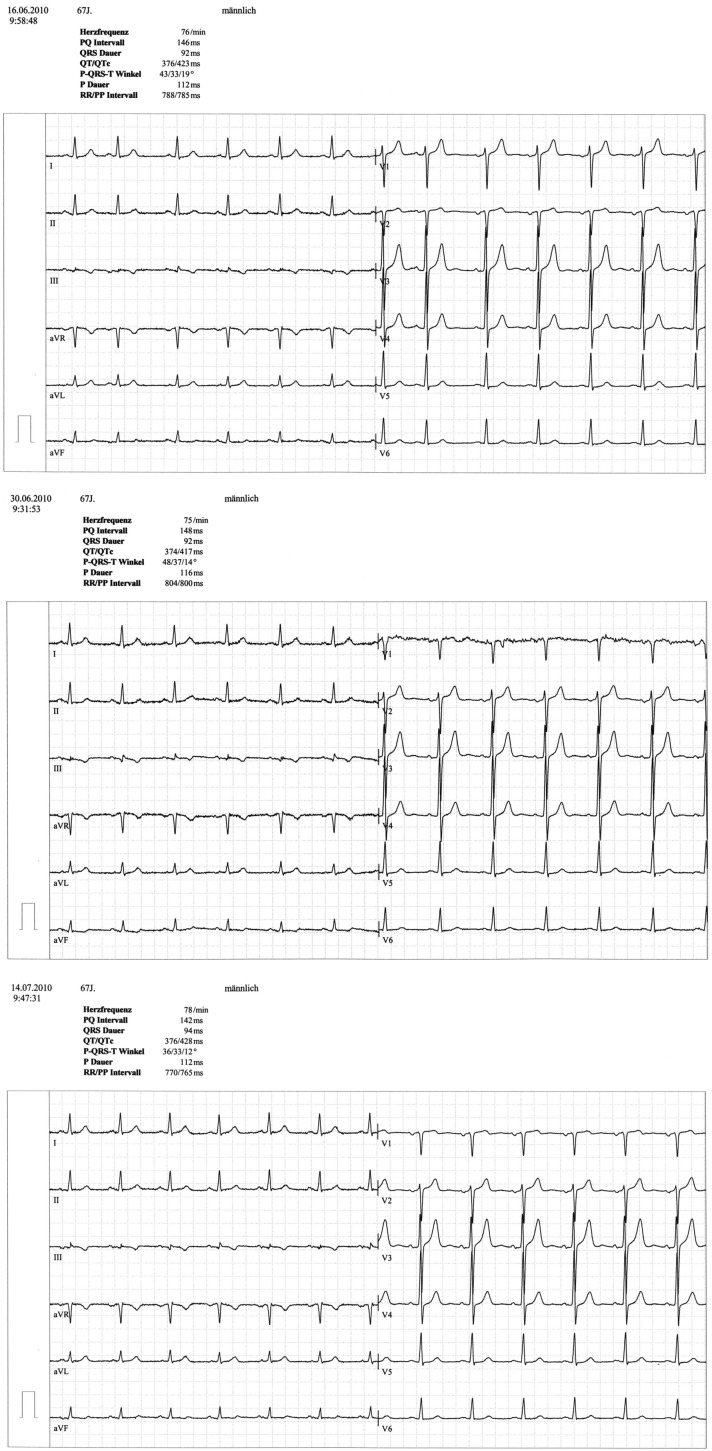
**ECG of patient 2 at baseline, week 2 and week 4 visit**.

**Figure 2:**
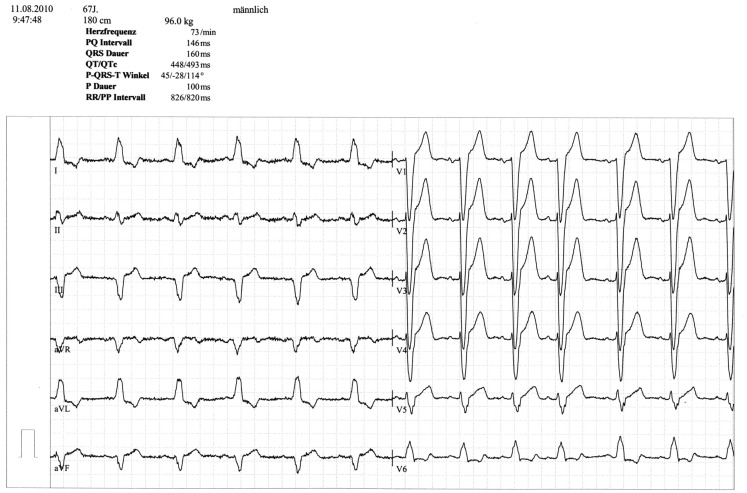
**ECG displaying LBBB at week 8 visit (patient 2)**.

**Figure 3:**
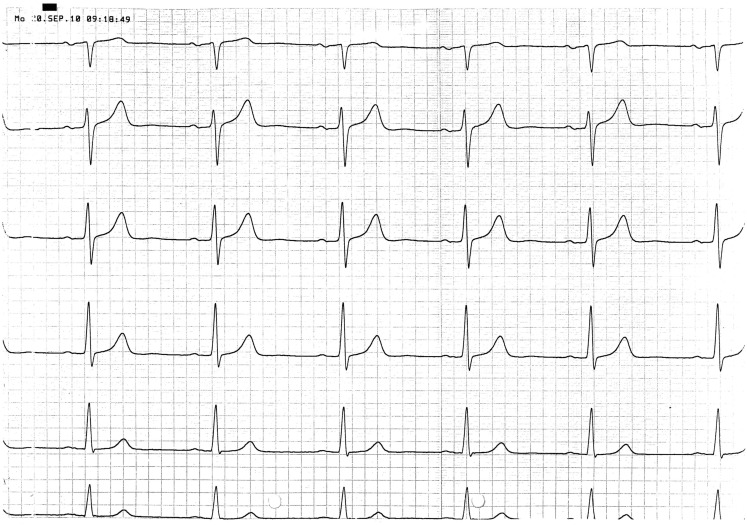
**ECG at follow-up 6 weeks after LBBB (patient 2)**.

A 67 year-old male patient (patient 2) displayed a LBBB with atrioventricular conduction time of 160 ms in a routine echocardiographic control performed after 8 weeks of tVNS, which had not been there at baseline and at the prior visits (screening/baseline, week 2, week 4). Prior echocardiography had not revealed any pathological results as shown in Figure 1. The patient himself did not experience any symptoms, the LBBB was detected in a routine control ECG according to the study protocol (see Figure 2). For improvement of mood and sleep he was on medication with mirtazapine (15 mg/day) and pre-existing arterial hypertension was treated with metoprolol 200 mg/day. Additional cardiac risk factors included smoking (10–15 cigarettes/day for almost 50 years), obesity (180 cm, 100 kg; BMI = 30.9 kg/m^2^), and hyperlipidemia. Extensive internal check-up including several ECG and sonographic examinations revealed a concentric hypertrophy of the left ventricle without regional cardiac movement disorders. Heart catheter examinations were recommended but refused by the patient. The LBBB was completely reversible, further ECG controls did not show similar episodes so far (see Figure 3). tVNS stimulation was immediately stopped after detecting the LBBB at week 8 study visit. This event was not classified as a severe adverse event as it did not lead to hospitalization of the patient or other grave impairment.

Further reported adverse events included headache (3×), breathing difficulties (3×), chest sensation (3×), dizziness (2×), subjective hearing impairment (2×), worsening of tinnitus, neck pain, croakiness, and sleeping disorder. Notably, all these complaints were reported to have been perceived transiently, they could not be objectified and no specific actions were taken. For none of these side effects there was a clear hint for a causal relationship to the intervention itself.

Side effects clearly related to the intervention were technical problems [contact problems (4×)] and local problems [local electrode pressure (11×); pain at stimulation with high intensity (3×)].

**Figure 4:**
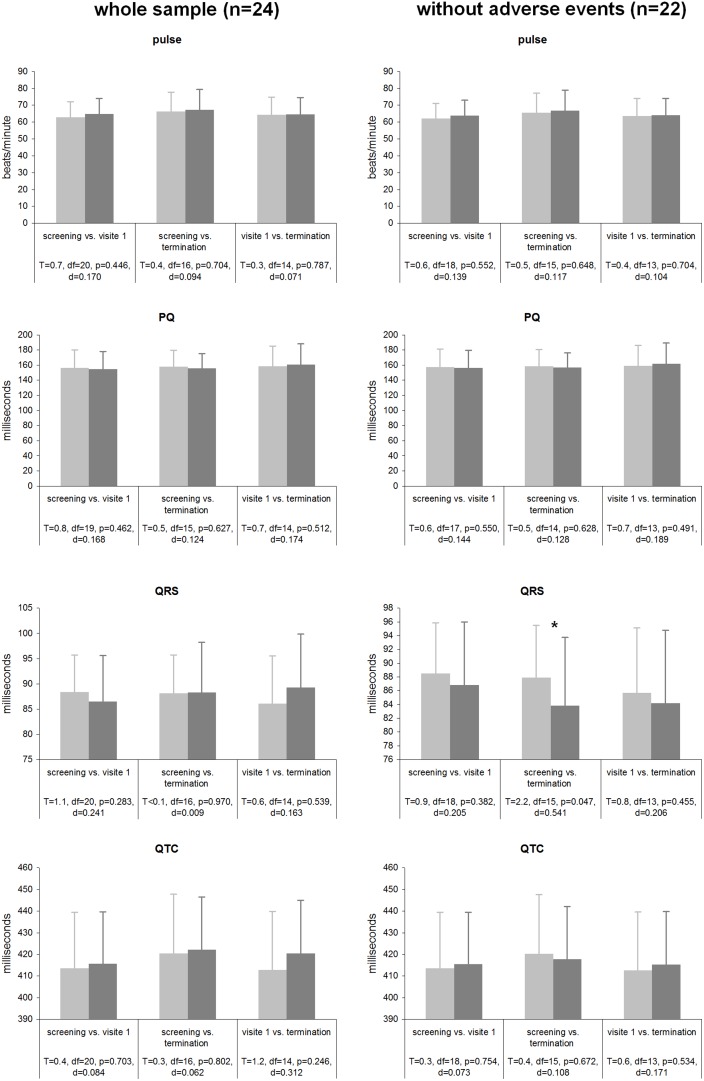
**Cardiac parameters during tVNS stimulation for both the whole sample and the sample without the 2 patients experiencing adverse cardiac events**.

Statistical analyses were performed on various ECG parameters [heart beats per minute, PQ interval (ms), QRS complex (ms), and QTc (ms)] both for the whole sample (*n* = 24) and the sample without the two patients with adverse events (*n* = 22; see Figure 4). For the whole sample none of the analyzed parameters showed significant differences between the three time points, effect sizes were small (all *p*s > 0.246; all *d*s < 0.312). When the two patients with cardiac averse events were excluded from analysis, a significant reduction of the QRS complex with a medium effect size (*p* = 0.047; *d* = 0.541) from screening to the termination emerged; all other contrasts were not significant with small effect sizes (all *p*s > 0.382; all *d*s < 0.206). All results are displayed in detail in Figure 4.

## Discussion

The purpose of this pilot study was to evaluate the feasibility and safety of tVNS in the treatment of chronic tinnitus. This is one of the first studies investigating feasibility and safety of tVNS in a clinical sample (Stefan et al., 2012). The main findings of our study were that (1) tVNS is feasible in tinnitus patients without any signs of long-term worsening of tinnitus complaints and that (2) in those patients without a history of known cardiac disease, these data suggest that tVNS cannot be considered unsafe. It was well tolerated in the majority of patients of this study sample. (3) With respect to the measured cardiac parameters, the study showed that tVNS of the left ABVN is unlikely to cause adverse cardiac reactions. In both of the reported cardiac adverse events other explanations for the symptoms were existing in the patients. Patient 1 had experienced sinusarrhythmic episodes already in the past, in patient 2 the comorbid condition of hypertension had caused concentric cardiac hypertrophy which might have contributed to the described temporary LBBB. In the statistical analysis of the whole sample no effects on ECG parameters were observed, after exclusion of the patients with side effects there was a significant *reduction* of the QRS time. However, this effect is small, and significance would not have survived correction for multiple testing. Thus it remains to be elucidated by further studies whether tVNS reduces the QRS time or whether the QRS time remains unaffected by tVNS. Definitively our data do not provide a hint for a prolongation of the QRS complex which is a known predictor of cardiac morbidity and mortality (Shamim et al., 1999; Kashani and Barold, 2005; Brenyo and Zareba, 2011).

The authors are well aware of the preliminary character of the presented data as the study was originally not designed for the assessment of *cardiac* effects of left-sided tVNS, but was rather conducted to examine feasibility and efficacy of tVNS in a sample of tinnitus patients. Therefore, the analyzed ECG recordings were performed based on standard safety considerations. Stable somatic comorbidities including cardiac diseases were not considered as a contraindication for participation in the study, unfortunately complicating the interpretation of the presented data *ex post*. Nonetheless, we would recommend a more conservative selection of patients in future clinical studies.

This is in line with the low incidence of adverse cardiac reactions during the long-term experience in more than 50,000 patients with implanted left VNS for treatment of epilepsy and depression (Ben-Menachem, 2002; Cristancho et al., 2011).

After intensive workup the two described cardiac adverse events during the study are more probably due to individual pre-existing cardiac risk factors such as known sinus-arrhythmias (patient 1), hypertensive state, and the attributable dilatative cardiomyopathy (patient 2).

Taking into consideration the relatively high age of our sample, the risk of internal somatic comorbidities is much higher than in general population samples and one should not prematurely judge the tVNS to be causally related to the adverse cardiac reactions described above.

Thus, our preliminary data do not provide concrete hints for relevant influences of tVNS on cardiac rhythm. In our opinion tVNS as approved by CE marking represents an innovative and promising new approach in non-invasive modulation of brain activity that might exert benefits in a variety of neuropsychiatric disorders and merits further research. Nevertheless, in light of the potential of tVNS to modulate conduction system of the heart, ECG recordings are recommended in every study of tVNS in order to obtain further safety data.

## Conflict of Interest Statement

The study was sponsored by CerboMed GmbH, Erlangen, Germany.

## Abbreviations

AV, atrioventricular; ECG, electrocardiography/electrocardiographic; LBBB, left bundle branch block; NTS, nucleus of the solitary tract/nucleus tractus solitarii; tVNS, transcutaneous vagus nerve stimulation; VNS, conventional vagus nerve stimulation.

## References

[B1] Ben-Menachem E. (2002). Vagus-nerve stimulation for the treatment of epilepsy. Lancet Neurol. 1, 477–482 10.1016/S1474-4422(02)00220-X 12849332

[B2] Brenyo A. Zareba W. (2011). Prognostic significance of QRS duration and morphology. Cardiol. J. 18, 8–17 21305480

[B3] Chae J. H. Nahas Z. Lomarev M. Denslow S. Lorberbaum J. P. Bohning D. E. George M. S. (2003). A review of functional neuroimaging studies of vagus nerve stimulation (VNS). J. Psychiatr. Res. 37, 443–455 10.1016/S0022-3956(03)00074-8 14563375

[B4] Cohen J. (1988). Statistical Power for the Behavioral Sciences. Hillsdale, NJ: Erlbaum

[B5] Cristancho P. Cristancho M. A. Baltuch G. H. Thase M. E. O’Reardon J. P. (2011). Effectiveness and safety of vagus nerve stimulation for severe treatment-resistant major depression in clinical practice after FDA approval: outcomes at 1 year. J. Clin. Psychiatry 72, 1376–1382 10.4088/JCP.09m05888blu 21295002

[B6] De Ferrari G. M. Crijns H. J. Borggrefe M. Milasinovic G. Smid J. Zabel M. Gavazzi A. Sanzo A. Dennert R. Kuschyk J. Raspopovic S. Klein H. Swedberg K. Schwartz P. J. (2011). Chronic vagus nerve stimulation: a new and promising therapeutic approach for chronic heart failure. Eur. Heart J. 32, 847–855 10.1093/eurheartj/ehq391 21030409

[B7] De Ferrari G. M. Sanzo A. Schwartz P. J. (2009). Chronic vagal stimulation in patients with congestive heart failure. Conf. Proc. IEEE Eng. Med. Biol. Soc. 2009, 2037–2039 19964772 10.1109/IEMBS.2009.5334414

[B8] Dietrich S. Smith J. Scherzinger C. Hofmann-Preiss K. Freitag T. Eisenkolb A. Ringler R. (2008). A novel transcutaneous vagus nerve stimulation leads to brainstem and cerebral activations measured by functional MRI. Biomed. Tech. (Berl.) 53, 104–111 10.1515/BMT.2008.022 18601618

[B9] Ellrich J. (2011). Transcutaneous vagus nerve stimulation. Eur. Neurol. Rev. 6, 262–264

[B10] Engineer N. D. Riley J. R. Seale J. D. Vrana W. A. Shetake J. A. Sudanagunta S. P. Borland M. S. Kilgard M. P. (2011). Reversing pathological neural activity using targeted plasticity. Nature 470, 101–104 10.1038/nature09656 21228773 PMC3295231

[B11] Grimm S. Bajbouj M. (2010). Efficacy of vagus nerve stimulation in the treatment of depression. Expert Rev. Neurother. 10, 87–92 10.1586/ern.09.138 20021323

[B12] Kashani A. Barold S. S. (2005). Significance of QRS complex duration in patients with heart failure. J. Am. Coll. Cardiol. 46, 2183–2192 16360044 10.1016/j.jacc.2005.01.071

[B13] Kosel M. Brockmann H. Frick C. Zobel A. Schlaepfer T. E. (2011). Chronic vagus nerve stimulation for treatment-resistant depression increases regional cerebral blood flow in the dorsolateral prefrontal cortex. Psychiatry Res. 191, 153–159 10.1016/j.pscychresns.2010.11.004 21306877

[B14] Kraus T. Hosl K. Kiess O. Schanze A. Kornhuber J. Forster C. (2007). BOLD fMRI deactivation of limbic and temporal brain structures and mood enhancing effect by transcutaneous vagus nerve stimulation. J. Neural Transm. 114, 1485–1493 10.1007/s00702-007-0755-z 17564758

[B15] Nemeroff C. B. Mayberg H. S. Krahl S. E. Mcnamara J. Frazer A. Henry T. R. George M. S. Charney D. S. Brannan S. K. (2006). VNS therapy in treatment-resistant depression: clinical evidence and putative neurobiological mechanisms. Neuropsychopharmacology 31, 1345–1355 10.1038/sj.npp.1301094 16641939

[B16] Pardo J. V. Sheikh S. A. Schwindt G. C. Lee J. T. Kuskowski M. A. Surerus C. Lewis S. M. Abuzzahab F. S. Adson D. E. Rittberg B. R. (2008). Chronic vagus nerve stimulation for treatment-resistant depression decreases resting ventromedial prefrontal glucose metabolism. Neuroimage 42, 879–889 10.1016/j.neuroimage.2008.04.267 18595737 PMC2601663

[B17] Peuker E. T. Filler T. J. (2002). The nerve supply of the human auricle. Clin. Anat. 15, 35–37 10.1002/ca.1089 11835542

[B18] Schachter S. C. Saper C. B. (1998). Vagus nerve stimulation. Epilepsia 39, 677–686 10.1111/j.1528-1157.1998.tb01151.x 9670894

[B19] Schwartz P. J. De Ferrari G. M. Sanzo A. Landolina M. Rordorf R. Raineri C. Campana C. Revera M. Ajmone-Marsan N. Tavazzi L. Odero A. (2008). Long term vagal stimulation in patients with advanced heart failure: first experience in man. Eur. J. Heart Fail. 10, 884–891 10.1016/j.ejheart.2008.07.016 18760668

[B20] Shamim W. Francis D. P. Yousufuddin M. Varney S. Pieopli M. F. Anker S. D. Coats A. J. (1999). Intraventricular conduction delay: a prognostic marker in chronic heart failure. Int. J. Cardiol. 70, 171–178 10.1016/S0167-5273(99)00077-7 10454306

[B21] Stefan H. Kreiselmeyer G. Kerling F. Kurzbuch K. Rauch C. Heers M. Kasper B. Hammen T. Rzonsa M. Pauli E. Ellrich J. Graf W. Hopfengaertner R. (2012). Transcutaneous vagus nerve stimulation (t-VNS) in pharmacoresistant epilepsies: a proof of concept trial. Epilepsia 53, e115–e118 10.1111/j.1528-1167.2012.03492.x 22554199

[B22] Vonck K. Boon P. Van Roost D. (2007). Anatomical and physiological basis and mechanism of action of neurostimulation for epilepsy. Acta Neurochir. Suppl. 97, 321–328 10.1007/978-3-211-33081-4_35 17691318

[B23] Vonck K. De Herdt V. Bosman T. Dedeurwaerdere S. Van Laere K. Boon P. (2008). Thalamic and limbic involvement in the mechanism of action of vagus nerve stimulation, a SPECT study. Seizure 17, 699–706 10.1016/j.seizure.2008.05.001 18556220

